# Functional analysis of the N‐terminal region of acetylxylan esterase from *Caldanaerobacter subterraneus* subsp. *tengcongensis*

**DOI:** 10.1002/2211-5463.13476

**Published:** 2022-09-20

**Authors:** Kohei Sasamoto, Tomoki Himiyama, Kunihiko Moriyoshi, Takashi Ohmoto, Koichi Uegaki, Tsutomu Nakamura, Yoshiaki Nishiya

**Affiliations:** ^1^ Division of Life Science, Graduate School of Science and Engineering Setsunan University Osaka Japan; ^2^ Biomedical Research Institute National Institute of Advanced Industrial Science and Technology Osaka Japan; ^3^ Osaka Research Institute of Industrial Science and Technology Japan; ^4^ Department of Applied Biological Chemistry, Faculty of Agriculture Kindai University Nara Japan; ^5^ Agricultural Technology and Innovation Research Institute Kindai University Nara Japan

**Keywords:** acetylxylan esterase, *Caldanaerobacter*, crystallization, intrinsically disordered region, protein solubility

## Abstract

Acetylxylan esterase from *Caldanaerobacter subterraneus* subsp. *tengcongensis* (TTE0866) has an N‐terminal region (NTR; residues 23–135) between the signal sequence (residues 1–22) and the catalytic domain (residues 136–324), which is of unknown function. Our previous study revealed the crystal structure of the wild‐type (WT) enzyme containing the NTR and the catalytic domain. Although the structure of the catalytic domain was successfully determined, that of the NTR was undetermined, as its electron density was unclear. In this study, we investigated the role of the NTR through functional and structural analyses of NTR truncation mutants. Based on sequence and secondary structure analyses, NTR was confirmed to be an intrinsically disordered region. The truncation of NTR significantly decreased the solubility of the proteins at low salt concentrations compared with that of the WT. The NTR‐truncated mutant easily crystallized in a conventional buffer solution. The crystal exhibited crystallographic properties comparable with those of the WT crystals suitable for structural determination. These results suggest that NTR plays a role in maintaining the solubility and inhibiting the crystallization of the catalytic domain.

## Graphical abstract

Acetylxylan esterase from *Caldanaerobacter subterraneus* subsp. *tengcongensis* (TTE0866) has a functionally unknown, intrinsically disordered N‐terminal region (NTR). In this article, we report that NTR plays a role in maintaining the solubility and inhibiting the crystallization of the catalytic domain. These findings provide new insights into the role of the NTR region in carbohydrate‐active enzymes.

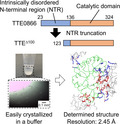

**Abbreviations**
AEX: anion‐exchange chromatography
AXE: acetylxylan esterase
BSA: bovine serum albumin
CA: cellulose acetate
CD: circular dichroism
CE: carbohydrate esterase
DS: degree of substitution
GlcNAc: *N*‐acetylglucosamine
HIC: hydrophobic interaction chromatography
IDR: intrinsically disordered region
imagej
: image processing and analysis in java
Ni‐NTA: nickel‐nitrilotriacetic acid affinity chromatography
NTR: N‐terminal region
PONDR: predictor of naturally disordered regions
SEC: size exclusion chromatography
WT: wild‐type

Acetylxylan esterase (AXE; EC3.1.1.72) hydrolyzes the acetyl groups from acetylated xylan [1, 2]. AXE has been proposed to be the initial enzyme in the biodegradation process of cellulose acetate (CA), a plastic used for membrane materials, by decreasing the degree of substitution (DS) by acetate [3, 4, 5].

We studied AXE from *Caldanaerobacter subterraneus* subsp. *tengcongensis* (a synonym for *Thermoanaerobacter tengcongensis* MB4; TTE0866) because this enzyme can uniquely deacetylate CA with a high DS of up to 2.45 [6]. TTE0866 is classified as a carbohydrate esterase family 4 (CE4) by carbohydrate‐active enzymes (CAZy), which include enzymes that hydrolyze *N*‐ or *O*‐acetyl groups of chitin and acetylxylan [7, 8, 9]. TTE0866 is composed of three regions: a signal sequence (residues 1–22), an N‐terminal region (NTR; residues 23–135), and a catalytic domain on the C‐terminus (CE4 domain; residues 136–324). The amino acid number of TTE0866 described in this report corresponds to the protein including the signal sequence. We previously determined the crystal structure of wild‐type TTE0866 (WT), containing residues 23–324 (the NTR and CE4 domains), to explain its high activity toward CA with a high DS [10]. Although the active site conformation of TTE0866 was highly similar to that of other CE4 enzymes, the orientation of the tryptophan residue (W264) near the active site was clearly distinct. The orientation of W264 formed a uniquely shaped cavity within TTE0866, which may contribute to its reactivity toward CA with a high DS. In contrast, in the crystallographic analysis, the electron density of NTR was not observed. This indicates that the NTR exhibits an undefined conformation or is truncated during crystallization.

In this study, we investigated the functional role of the NTR of TTE0866. The NTR of TTE0866 has several characteristics, as suggested by sequence analysis [10]. The NTR is mostly comprised of hydrophilic and charged amino acid residues. The NTR was homologous to eight proteins, with approximately 30% homology. All retrieved sequences were from the region adjacent to the CE4 homologous region, and seven were from the genus *Thermoanaerobacter*. Despite these similarities, their functions have not yet been elucidated. Additionally, a CE4 enzyme with a functionally unknown disordered N‐terminal region has been reported [11]. Our study revealed the importance of the NTR in maintaining protein solubility.

## Materials and methods

### Preparation of WT, TTE^Δ20^, TTE^Δ40^, TTE^Δ52^, TTE^Δ60^, TTE^Δ80^, TTE^Δ100^, and TTE^Δ110^

To examine the role of NTR, we prepared truncation mutants: TTE^Δ20^, TTE^Δ40^, TTE^Δ52^, TTE^Δ60^, TTE^Δ80^, TTE^Δ100^, and TTE^Δ110^, in which the N‐terminal amino acid residues were truncated by 20, 40, 52, 60, 80, 100, and 110, respectively (Fig. S1). The gene encoding TTE0866 was cloned into the pET‐32b plasmid vector (Novagen, Madison, WI, USA). PCR amplification was performed using a Veriti200 thermal cycler (Thermo Fisher Scientific, Waltham, MA, USA). Site‐directed mutagenesis was performed using a PrimeSTAR mutagenesis kit (Takara Bio, Shiga, Japan) and a KOD Plus mutagenesis kit (Toyobo, Osaka, Japan). The primers used are listed in Table S1. *E.* Rosetta2 (DE3) pLysS cells (Novagen) harboring the expression plasmid were cultured in Luria‐Bertani (LB) medium, and expression of the gene was induced by isopropyl β‐D‐1‐thiogalactopyranoside (IPTG). Purification of WT, TTE^Δ20^, TTE^Δ40^, TTE^Δ52^, and TTE^Δ60^ was performed as previously described [10]. Purification of TTE^Δ80^, TTE^Δ100^, and TTE^Δ110^ was performed by a slightly modified procedure using hydrophobic interaction chromatography (HIC) with a HiTrap Butyl HP column (Cytiva, Tokyo, Japan) instead of anion‐exchange chromatography (AEX). Protein purity was confirmed by SDS/PAGE. Protein concentrations were determined by measuring absorbance at 280 nm using a NanoDrop2000 UV–Vis spectrometer (Thermo Fisher Scientific). Molecular weight (molar extinction coefficient; ε) of TTE^Δ20^, TTE^Δ40^, TTE^Δ52^, TTE^Δ60^, TTE^Δ80^, TTE^Δ100^, and TTE^Δ110^ are 34 510 (26 740 m ^−1^·cm^−1^), 32 010 (21 620 m ^−1^·cm^−1^), 29 849 (21 620 m ^−1^·cm^−1^), 28 373 (20 340 m ^−1^·cm^−1^), 27 588 (20 340 m ^−1^·cm^−1^), 25 299 (20 340 m ^−1^·cm^−1^), 22 941 (20 340 m ^−1^·cm^−1^), and 21 908 (20 340 m ^−1^·cm^−1^), respectively.

### Preparation of WT and TTE^Δ100^ for enzymatic reactions

We previously found that the active site of WT was occupied by Ni^2+^ ions taken up by nickel‐nitrilotriacetic acid affinity chromatography (Ni‐NTA) during purification [10]. Therefore, to investigate metal dependency, we avoided the use of Ni‐NTA to purify WT and TTE^Δ100^.

Proteins from sonicated and heat‐treated solutions of recombinant *E. coli* were separated by AEX on a HiTrap Q column (Cytiva). The fusion tag was cleaved using PreScission protease (Takara Bio). Separation of the WT and the fusion tag was performed by AEX using a HiTrap Q column. The final purification step was size‐exclusion chromatography (SEC) using a HiLoad 26/600 Superdex 75 prep‐grade column (Cytiva).

Proteins from sonicated and heat‐treated solutions of recombinant *E. coli* were precipitated by a 40% saturation concentration of the ammonium sulfate fraction. The next purification step was AEX, using a HiTrap Q column. The separation of TTE^Δ100^ and the fusion tag was performed by HIC using a HiTrap Butyl HP column. The final purification step was SEC using a HiLoad 26/600 Superdex 75 prep‐grade column. Purified WT and TTE^Δ100^ were dialyzed overnight in the presence of 50 mm EDTA to remove metal ions, and the solvent was substituted with 50 mm sodium phosphate buffer (pH 8.0) containing 150 mm NaCl.

### Preparation of NTR fragment

For expression of the NTR fragment, we used the N^pro^(EDDIE) expression system, a mutant of the N‐terminal auto‐protease from classical swine fever virus [12]. The gene encoding N^pro^(EDDIE)‐NTR was cloned into the pET‐11a plasmid vector (Novagen) using an In‐Fusion HD cloning kit (Takara Bio). *E. coli* BL21 (DE3) pLysS cells (Novagen) harboring the expression plasmid were cultured in LB medium, and gene expression was induced by IPTG. Subsequent purification using the N^pro^(EDDIE) expression system followed the procedures described by Goda et al. [13]. The final purification step was AEX, using a HiTrap Q column. Protein purity was confirmed by SDS/PAGE. MALDI‐TOF MS measurements, using a Bruker Autoflex III mass spectrometer, showed that the molecular weight of the NTR fragment was 13 039, which matched well with the molecular weight calculated from the amino acid sequence (13 076). Protein concentrations were determined by measuring absorbance at 280 nm using a NanoDrop2000 UV–Vis spectrometer [molecular weight 13 076; molar extinction coefficient (ε) = 6400 m ^−1^·cm^−1^].

### Circular dichroism (CD)

The CD spectra were obtained on a J‐820 spectropolarimeter (Jasco, Tokyo, Japan) equipped with a Jasco PTC‐423L temperature controller using a 1 mm cuvette. The protein solution (0.2 g·L^−1^) contained 20 mm Tris–HCl buffer (pH 8.0) and 150 mm NaCl. *T*_m_‐values were calculated by measuring the CD signals at 222 nm from 20 to 90 °C.

### Limited proteolysis

Proteolysis of NTR fragment and bovine serum albumin (BSA) was performed using trypsin digestion at 25 °C in 15 mm sodium phosphate buffer (pH 7.0) and 50 mm NaCl. For 4 μg of trypsin, 265 μg of the NTR fragment or 50 μg of BSA was used. Proteolysis was stopped by the addition of 10% acetic acid. Digestion was examined by separating the protein fragments using SDS/PAGE.

### Size exclusion chromatography

Analytical SEC was performed using an AKTA FPLC equipped with a Superdex 75 Increase 10/300 GL column (Cytiva), where 100 μL of protein solution (1.0 mg·mL^−1^) was applied to the column and eluted with 20 mm Tris–HCl (pH 8.0) buffer containing 150 mm NaCl. The UV absorption at 280 nm was monitored during the measurements.

### Enzymatic activity

*N*‐acetylglucosamine (GlcNAc) was purchased from Nacalai Tesque (Kyoto, Japan). (GlcNAc)_2–5_ were purchased from Tokyo Chemical Industry (Tokyo, Japan). (GlcNAc)_6_ was purchased from MegaZyme (Bray, Ireland). The reactions were conducted using 2 mm substrate, 5 μm metal chloride, and 1 μm enzyme in 50 mm potassium phosphate buffer (pH 8.0) and 150 mm NaCl. The reaction mixture was incubated at 60 °C. The carboxylic acid concentrations were determined three times every 10 min based on the procedure reported by Sakai et al. [14] using a standard curve plotted against the standard acetate (0–0.2 mm).

### Protein solubility

The solubility of WT, TTE^Δ20^, TTE^Δ40^, TTE^Δ52^, TTE^Δ60^, TTE^Δ80^, TTE^Δ100^, and TTE^Δ110^ to the buffers was evaluated by quantifying the soluble protein concentration after dialysis against 20 mm Tris–HCl (pH 8.0) buffer with 0 or 150 mm NaCl at 10 °C for 16 h.

After dissolving the ammonium sulfate precipitate of WT and TTE^Δ100^ into 20 mm Tris–HCl (pH 8.0) buffer containing 100 mm NaCl, 100 μL of protein solution (100 μm) was dialyzed against 20 mm sodium phosphate (pH 6.0) buffer, 20 mm Tris–HCl (pH 8.0) buffer, or 20 mm CHES‐NaOH (pH 10.0) buffer containing 0, 50, 100, and 150 mm NaCl at 10 °C for 16 h. The dialyzed solutions were centrifuged at 20 000 ***g*** for 30 min. The supernatant volume was adjusted to 100 μL using a microsyringe. Protein concentrations were determined by measuring absorbance at 280 nm using a NanoDrop2000 UV–Vis spectrometer. The protein concentrations were averaged over three independent experiments.

To check the effect of NTR on TTE^Δ100^ solubility, 200 μm TTE^Δ100^ (50 μL) and 0, 100, 200, 300, and 400 μm NTR fragments (50 μL) were mixed in 20 mm Tris–HCl (pH 8.0) buffer containing 100 mm NaCl. The protein solution was dialyzed against 20 mm Tris–HCl (pH 8.0) buffer at 10 °C for 16 h. The dialyzed proteins were centrifuged at 20 000 ***g*** for 30 min. The supernatant volume was adjusted to 100 μL using a microsyringe. The amount of soluble TTE^Δ100^ was quantified by SDS/PAGE band intensity using image processing and analysis in the java (imagej) program [15].

### Crystallization and structure determination

After dissolving the ammonium sulfate precipitation of TTE^Δ100^ into 20 mm Tris–HCl (pH 8.0) buffer containing 100 mm NaCl conditions, the protein solution (9.0 mg·mL^−1^) was dialyzed against 20 mm Tris–HCl (pH 8.0) buffer containing 100 mm NaCl. The solution obtained was incubated at room temperature for 3 days to generate protein crystals. Prior to flash‐cooling in a nitrogen flow at −180 °C, the crystals were cryoprotected with 20 mm Tris–HCl (pH 8.0) buffer, 100 mm NaCl, and 25% glycerol.

Diffraction data were collected on the beamline BL45XU at SPring‐8 (Hyogo, Japan) using the zoo program [16]. Diffraction data from multiple crystals were merged using the kamo program [17]. The diffraction data were integrated using the xds package [18, 19]. Scaling was performed using the aimless program [20]. The initial phase of the crystal was determined by molecular replacement using molrep from the CCP4 suite based on the crystal structure of TTE0866WT (Protein Data Bank (PDB) ID: 7FBW) [21]. Structural refinement was performed using REFMAC5 from the CCP4 suite [22].

## Results and discussion

### Structural analysis of NTR

**Fig. 1:**
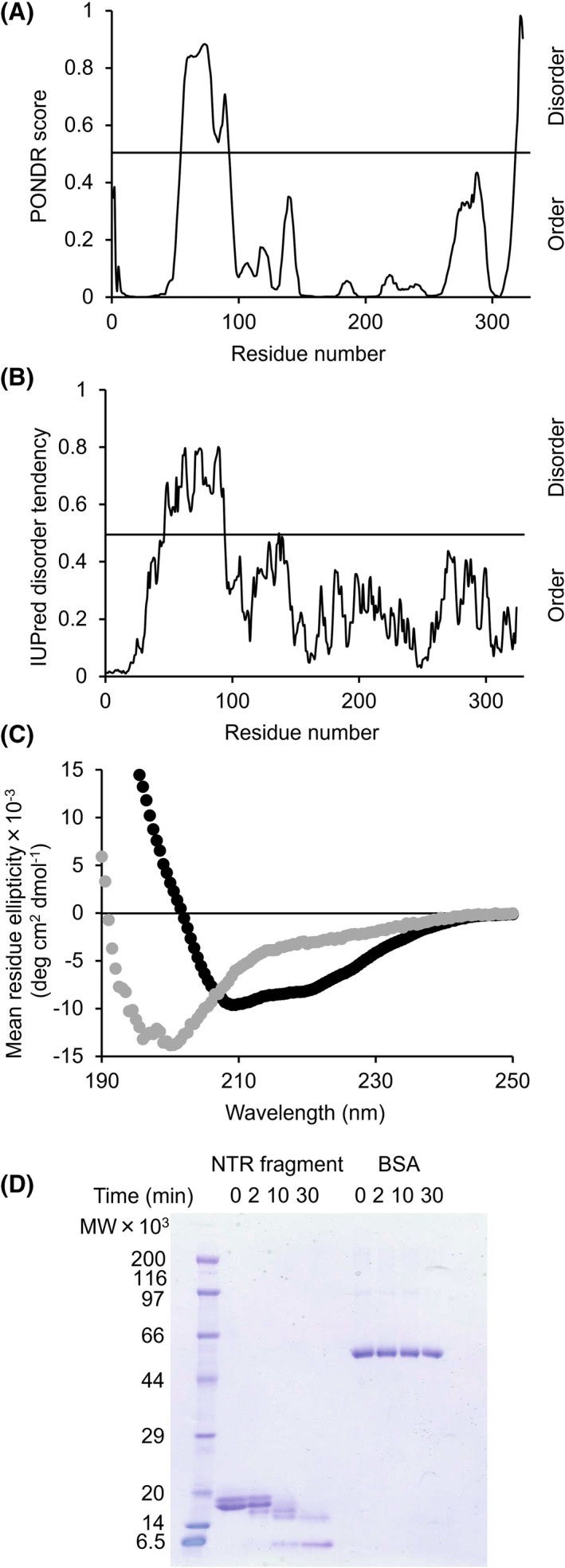
Properties of NTR. Disorder predictions of TTE0866 by (A) PONDR and (B) IUPred. Predicted disordered regions are the region above 0.5. (C) CD spectra of WT (black) and NTR (gray). (D) NTR or BSA were incubated with trypsin for the indicated times. Proteins were separated by SDS/PAGE and visualized by Coomassie brilliant blue.

We examined the sequence characteristics of NTR using predictor of naturally disordered regions (PONDR) and IUPred [23, 24]. The NTR had a low‐homology repeated sequence (Fig. S2). The NTR exhibits sequence identity only to the region adjacent to the CE4 homologous domain from the genus *Thermoanaerobacter* (approximately 30% homology), which is a highly hydrophilic region [10]. However, the amino acid sequence of the NTR did not match any functionally annotated proteins. Next, we applied multiple protein disorder prediction methods to the NTR sequences. The disorder prediction in Fig. 1A was made using PONDR, which predicts disorder based on sequence attributes typically found in regions that are absent in X‐ray and nuclear magnetic resonance structures [23]. The results showed that TTE0866 was disordered in Asn55 to Asn92, located in the NTR. The disorder prediction shown in Fig. 1B was made using IUPred, which predicts disorder by analyzing pairwise energies between local protein sequences and predicting the likelihood that they will form globular structures [24]. The results showed that TTE0866 was disordered from Leu47 to Asp94 located on the NTR, which matched well with the results obtained in PONDR. The residues 23–46 were confirmed to be a highly hydrophilic region similar to the residues 47–94 by the hydropathy plot described in the previous study [10]. Thus, the majority of the NTR (72 of 113 residues, 64%) is a disordered region.

We examined the secondary structure of NTR using circular dichroism (CD). CD spectra of WT (residues 23–324) and NTR fragment (residues 23–137) are shown in Fig. 1C. The WT exhibited ellipticity minima at 210 and 220 nm, typical of proteins composed of α‐helices and β‐strands. By contrast, the NTR fragment exhibited a single minimum at 200 nm, typical of unstructured conformation [25, 26]. The spectral shape was similar to the CD spectrum with 60% random coils in the sequence [27]. The percentage of random coils was corresponding to the percentage indicated as a disordered region in the prediction (64%). We conducted limited proteolysis for the NTR fragment, based on the finding that protease cleavage is more likely to occur in disordered regions than in structured regions [28, 29]. Bovine serum albumin (BSA) was used as the control. The NTR fragment and BSA were exposed to trypsin for 2, 10, and 30 min and analyzed using SDS/PAGE. The NTR fragment was partially or completely digested, whereas BSA resisted digestion throughout the time course of the protease treatment (Fig. 1D). The results of sequence analysis, CD spectra, and limited proteolysis confirmed that the NTR holds the known characteristics of intrinsically disordered regions (IDRs) [30, 31]. Although many IDRs for hydrolases have been found, the average lengths of IDRs are 44 and 12 amino acids for eukaryotes and bacteria, respectively [32]. The percentages of proteins with IDRs of 30 or more residues were 2.0%, 4.2%, and 33.0% for archaea, eubacteria, and eukaryotes, respectively [33]. Thus, the length of the NTR (113 amino acids) is unique among IDRs found within bacteria. To the best of our knowledge, this is the first example of an IDR present in an extracellular enzyme from a thermophilic anaerobic bacterium.

### Properties of the NTR‐truncated mutants

**Fig. 2:**
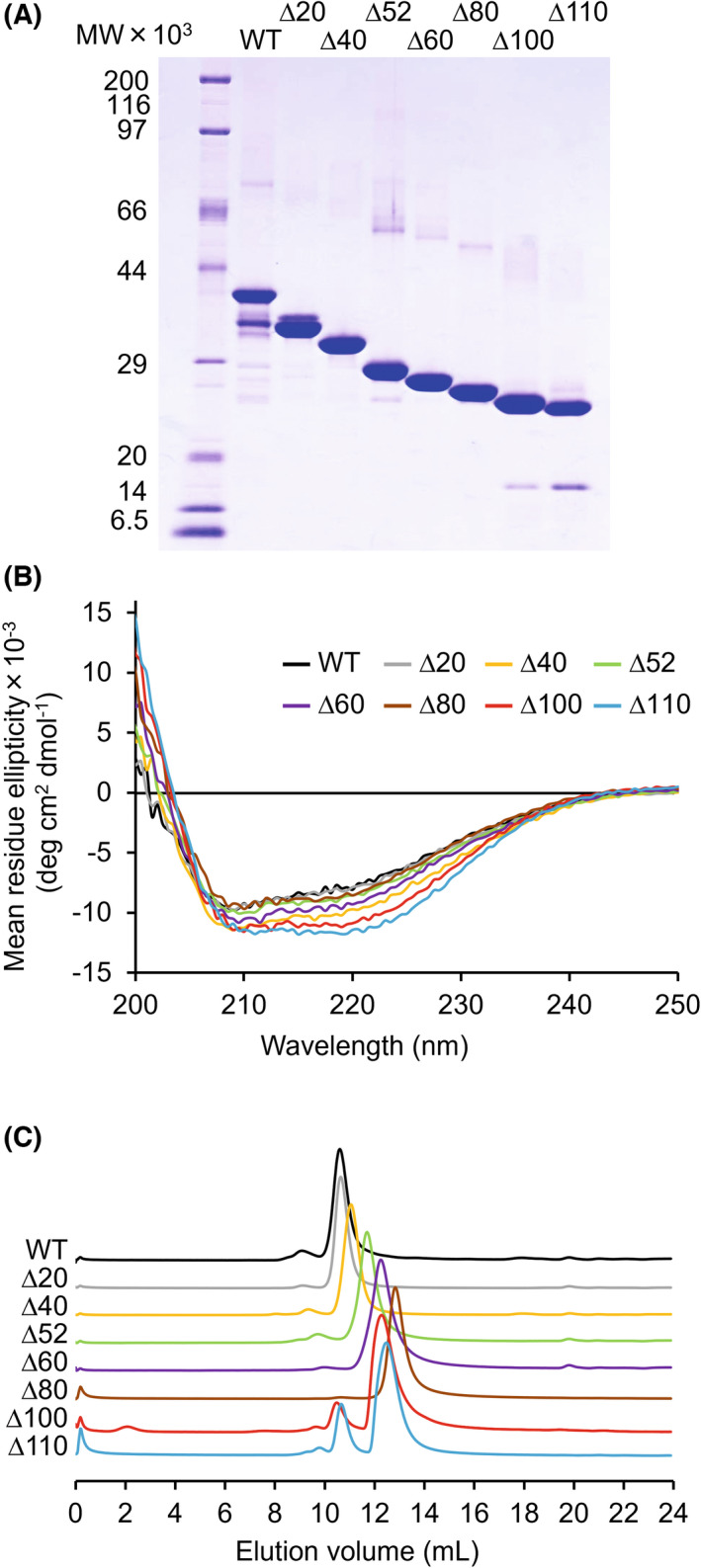
Properties of NTR truncation mutants. (A) SDS/PAGE for WT, TTE^Δ20^, TTE^Δ40^, TTE^Δ52^, TTE^Δ60^, TTE^Δ80^, TTE^Δ100^, and TTE^Δ110^. (B) CD spectra and (C) SEC analysis. SECs were normalized to the peak tops of each sample. Samples: WT (black), TTE^Δ20^ (gray), TTE^Δ40^ (yellow), TTE^Δ52^ (green), TTE^Δ60^ (purple), TTE^Δ80^ (brown), TTE^Δ100^ (red), and TTE^Δ110^ (blue).

To examine the role of NTR, we investigated the stability against temperature and denaturation for WT, TTE^Δ20^, TTE^Δ40^, TTE^Δ52^, TTE^Δ60^, TTE^Δ80^, TTE^Δ100^, and TTE^Δ110^ based on secondary structure analyses using CD spectra. WT, TTE^Δ20^, TTE^Δ40^, TTE^Δ52^, TTE^Δ60^, TTE^Δ80^, TTE^Δ100^, and TTE^Δ110^ were successfully obtained (Fig. 2A). SDS/PAGE of these truncation mutants revealed a stepwise molecular weight decrease resulting from truncation of the NTR. The CD spectra of WT, TTE^Δ20^, TTE^Δ40^, TTE^Δ52^, TTE^Δ60^, TTE^Δ80^, TTE^Δ100^, and TTE^Δ110^ suggested that the secondary structure content increased as the truncated NTR regions were extended (Fig. 2B). *T*_m_‐values of WT, TTE^Δ100^, and TTE^Δ110^ were estimated as 78.4, 76.8, and 78.4 °C, respectively (Fig. S3). These results suggest that thermostability is not significantly affected by NTR truncation.

To understand the effect of NTR truncation on the solution structure, the elution volumes of WT, TTE^Δ20^, TTE^Δ40^, TTE^Δ52^, TTE^Δ60^, and TTE^Δ80^ were investigated using analytical SEC. The elution volumes of WT, TTE^Δ20^, TTE^Δ40^, TTE^Δ52^, TTE^Δ60^, and TTE^Δ80^ were respectively observed at 10.6, 10.7, 11.0, 11.7, 12.2, and 12.8 mL (Fig. 2C). WT, TTE^Δ20^, TTE^Δ40^, TTE^Δ52^, TTE^Δ60^, and TTE^Δ80^ exhibited single peaks corresponding to molecular weights of 44 000, 43 000, 38 000, 31 000, 26 000, and 20 000, respectively, based on the calibration standard. The molecular weights of the monomers calculated from the amino acid sequences of WT, TTE^Δ20^, TTE^Δ40^, TTE^Δ52^, TTE^Δ60^, and TTE^Δ80^ were 34 000, 32 000, 30 000, 28 000, 27 000, and 25 000, respectively, roughly matching the estimated values. On the contrary, TTE^Δ100^ and TTE^Δ110^ gave two peaks: 10.5 and 12.3 mL for TTE^Δ100^, and 10.7 and 12.5 mL for TTE^Δ110^. The elution volume of the first peaks of TTE^Δ100^ and TTE^Δ110^ corresponded to molecular weights of 46 000 and 43 000, respectively, and the second peaks of TTE^Δ100^ and TTE^Δ110^ corresponded to molecular weights of 25 000 and 23 000, respectively, based on the calibration standard. The molecular weights of the monomers, calculated from the amino acid sequences of TTE^Δ100^ and TTE^Δ110^, were 23 000 and 22 000, respectively. The first and second peaks of TTE^Δ100^ and TTE^Δ110^ were expected to be dimers and monomers, respectively. Based on the peak area, the dimer‐to‐monomer percentage of TTE^Δ100^ was 10% and 90%, and the dimer‐to‐monomer percentage of TTE^Δ110^ was 20% and 80%, respectively. These results suggest that complete truncation of the NTR causes dimerization of the protein.

### Enzyme activities of the WT and NTR‐truncated mutant

The effect of NTR on the enzymatic activity was investigated by comparing the catalytic activities of WT and TTE^Δ100^. (GlcNAc)_1–6_ were used as substrates, and the released carboxylate (acetate) ions were monitored by chemical methods [14].

Most functionally determined CE4 enzymes use divalent metal ions such as Co^2+^, Ni^2+^, and Zn^2+^ [34, 35, 36, 37]. Co^2+^, Ni^2+^, and Zn^2+^ were added to the WT reaction solution and the enzymatic activity toward (GlcNAc)_3_ was measured. The results showed that the specific activity of WT was 0.027 ± 0.005 U·mg^−1^. The specific activities in the presence of Co^2+^, Ni^2+^, and Zn^2+^ were 0.26 ± 0.01, 0.22 ± 0.01, and 0.094 ± 0.014 U·mg^−1^, respectively. Thus, the optimum metal ion for TTE0866 was Co^2+^.

The specific activities of WT and TTE^Δ100^ toward (GlcNAc)_2–6_ are shown in Table 1. WT and TTE^Δ100^ were inactive toward the GlcNAc monomer. The specific activities of the WT and TTE^Δ100^ increased as the chain length of the substrate increased from (GlcNAc)_2_ to (GlcNAc)_6_. Although the specific activity of TTE^Δ100^ for each substrate was decreased by 54–83% compared with the WT, the removal of the NTR region did not completely lose its catalytic activity.

**Table 1:** Substrate specificity of WT and TTE^Δ100^. The substrate was incubated with 1 μm WT and TTE^Δ100^ for three times every 10 min at 60 °C, and released acetic acid was measured using chemical methods. One unit means the amount of enzyme that produces 1 μm·min^−1^ products. The data were averaged over three independent experiments.

	Specific activity (U·mg^−1^)				
	(GlcNAc)_2_	(GlcNAc)_3_	(GlcNAc)_4_	(GlcNAc)_5_	(GlcNAc)_6_
WT	0.075 ± 0.002	0.27 ± 0.01	1.1 ± 0.1	2.2 ± 0.1	2.8 ± 0.1
TTE^Δ100^	0.018 ± 0.015	0.045 ± 0.010	0.35 ± 0.05	0.81 ± 0.02	1.3 ± 0.1

### Protein solubilization by NTR

The solubilities of NTR‐truncated mutants were examined. Ammonium sulfate precipitates of the WT, TTE^Δ20^, TTE^Δ40^, TTE^Δ52^, TTE^Δ60^, TTE^Δ80^, TTE^Δ100^, and TTE^Δ110^ mutants were dissolved in 20 mm Tris–HCl (pH 8.0) buffer containing 150 mm NaCl. The WT, TTE^Δ20^, TTE^Δ40^, TTE^Δ52^, TTE^Δ60^, and TTE^Δ80^ mutants were completely dissolved even after removing NaCl by desalting the salt, whereas TTE^Δ100^ and TTE^Δ110^ mutants formed precipitates and the protein concentration in the solution decreased significantly.

**Fig. 3:**
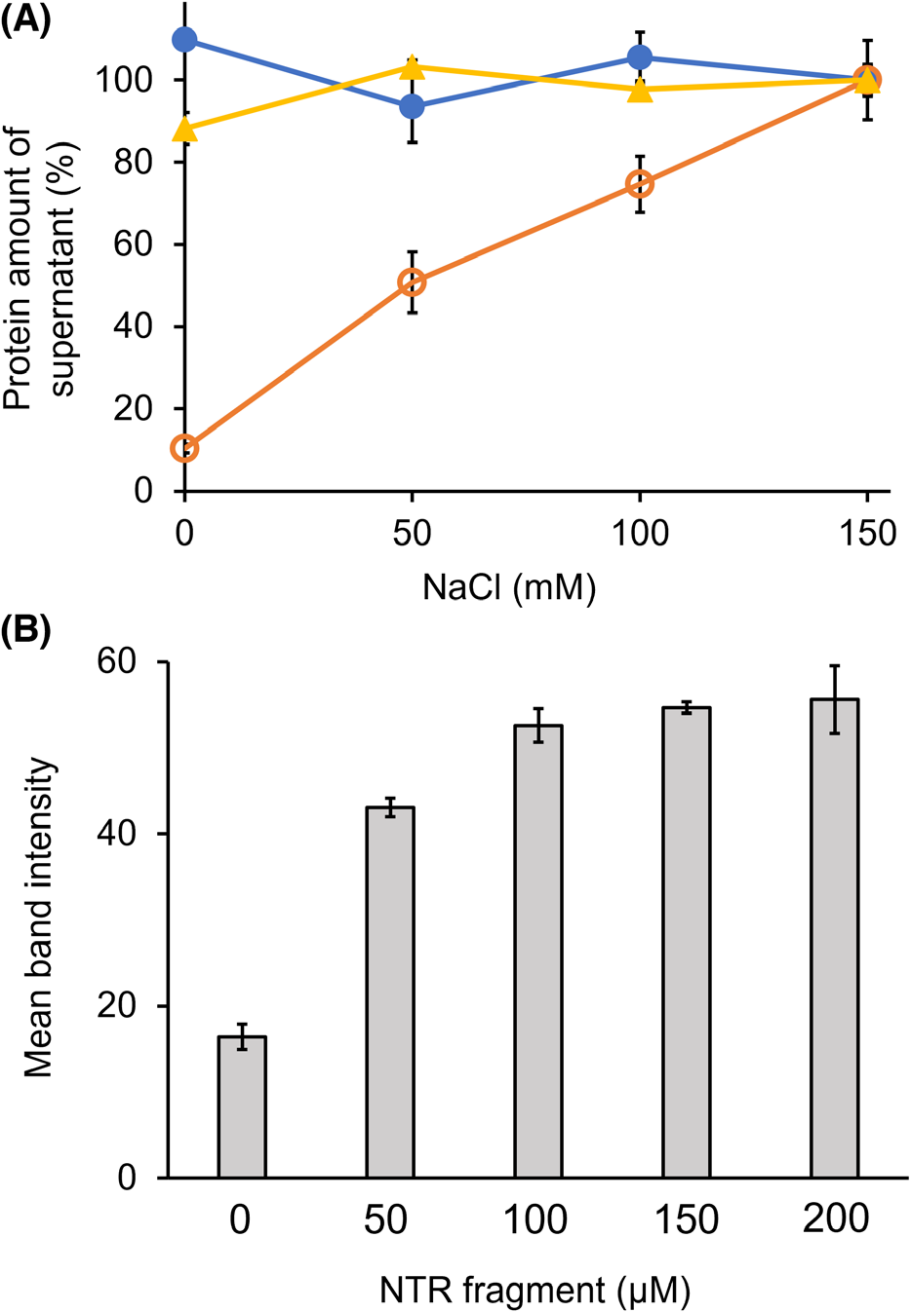
Protein solubilities of WT and TTE^Δ100^. (A) Protein concentration of the WT and TTE^Δ100^ under 20 mm Tris–HCl (pH 8.0) containing 0, 50, 100, and 150 mm NaCl. Samples: 100 μm WT (closed circles), 100 μm TTE^Δ100^ (open circles), and 100 μm TTE^Δ100^ with 100 μm NTR fragment (triangles). (B) The solubility of TTE^Δ100^ (100 μm) in the presence of NTR fragment (0, 50, 100, 150, and 200 μm) in 20 mm Tris–HCl (pH 8.0). Proteins were separated by SDS/PAGE and visualized by Coomassie brilliant blue (Fig. S5). The band intensity was evaluated by imagej program. Values are presented as the means ± SD (*n* = 3).

WT and TTE^Δ100^ were dialyzed for 16 h in 20 mm Tris–HCl (pH 8.0) buffer containing 0, 50, 100, and 150 mm NaCl. Soluble proteins at each salt concentration are shown in Fig. 3A. The supernatant protein concentration, when dialyzed in 150 mm NaCl, was set to 100%. The solubility of WT did not change with salt concentration, suggesting high solubility of WT. By contrast, the solubility of TTE^Δ100^ was strongly dependent on the NaCl concentration, which was significantly lower at 0 m NaCl. The experiment was also performed using 20 mm sodium phosphate buffer (pH 6.0) and 20 mm CHES‐NaOH (pH 10.0) buffer (Figs S4A,B). The results showed that WT was completely dissolved at pH 6.0 and pH 10.0, without being affected by the NaCl concentration. TTE^Δ100^ showed the lowest solubility with 0 m NaCl at pH 6.0 and pH 10.0, similar to the tendency at pH 8.0.

The solubility of TTE^Δ100^ was improved by addition of the NTR fragment (Fig. 3B and Fig. S5). NTR fragments (0, 50, 100, 150, and 200 μm) were added to 100 μm TTE^Δ100^ in 20 mm Tris–HCl buffer (pH 8.0). The solubility of TTE^Δ100^ was comparable with that of WT when an equal molar amount of NTR fragment was added to TTE^Δ100^ (Fig. 3A). This indicates that covalent linking of the NTR fragment is not necessary to dissolve the catalytic domain of TTE0866.

TTE^Δ100^ formed precipitates at low salt concentrations. Precipitation was observed in 20 mm sodium phosphate (pH 6.0), 20 mm Tris–HCl (pH 8.0), and 20 mm CHES‐NaOH (pH 10.0) buffers. The precipitates were crystalline (Fig. S6). TTE^Δ100^ dissolved in 20 mm sodium phosphate buffer (pH 6.0), 20 mm Tris–HCl (pH 8.0), and 20 mm CHES‐NaOH (pH 10.0) buffer containing 100 mm NaCl were crystallized during storage at room temperature for 3 days. Comparing the crystal sizes of the 0 m and 100 mm NaCl conditions, a large number of small crystals were observed under the low salt concentration, whereas relatively large crystals were observed under the 100 mm NaCl conditions.

### Structure determination of NTR‐truncated mutant

**Fig. 4:**
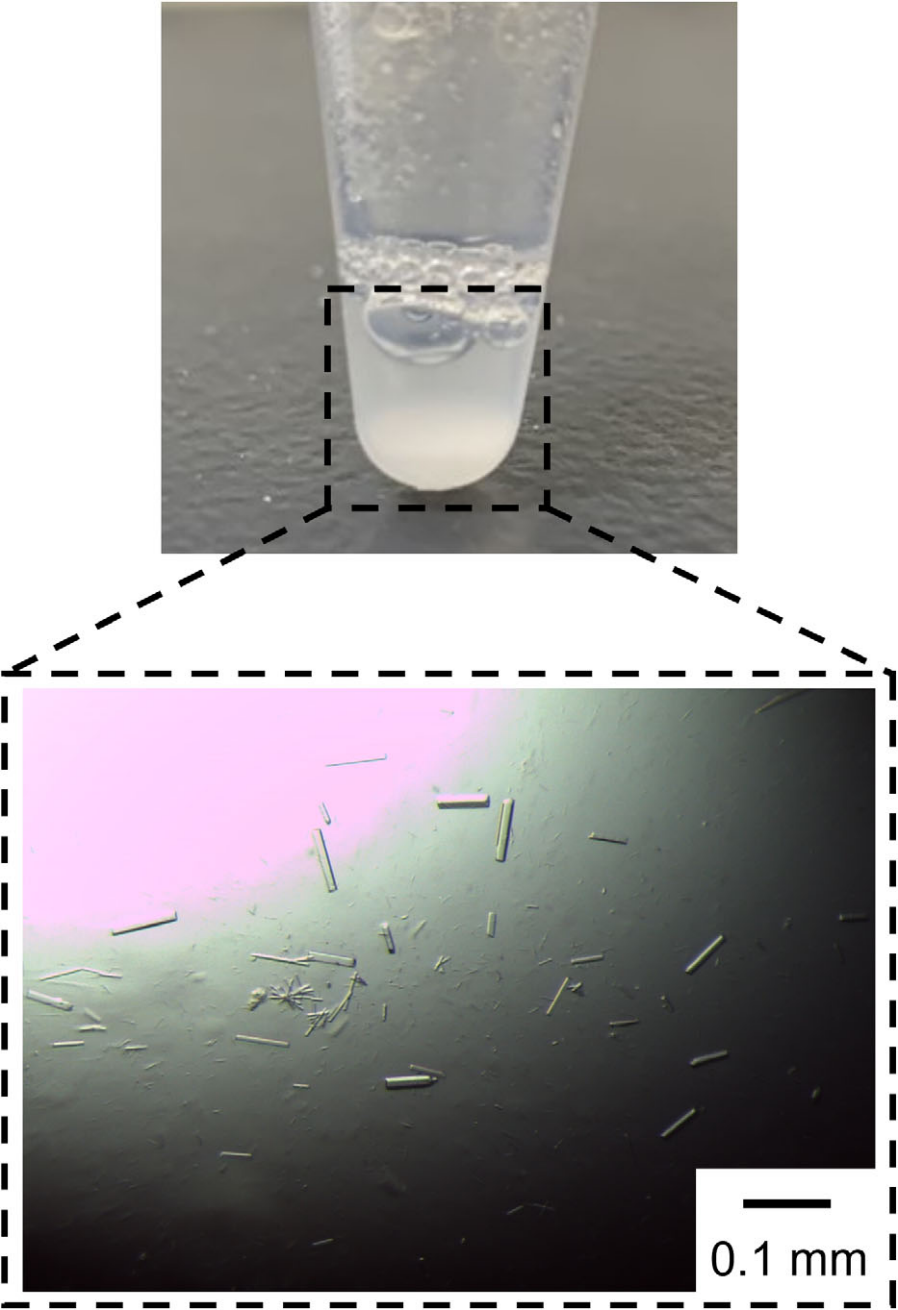
Crystallization of TTE^Δ100^. Precipitation formed during storage at room temperature for 3 days. Scale bars = 0.1 mm.

When the protein concentration was increased to promote crystallization, TTE^Δ100^ (9.0 mg·mL^−1^) dissolved in 20 mm Tris–HCl (pH 8.0) buffer containing 100 mm NaCl crystallized during storage at room temperature for 3 days (Fig. 4). X‐ray crystallographic analysis was performed using the crystalline particles. We successfully obtained the crystal structure of TTE^Δ100^. The TTE^Δ100^ crystal diffracted to 2.45 Å resolution and belonged to the space group *I*2_1_2_1_2_1_. The initial phase was determined by molecular replacement. The crystal structure was refined to *R*_cryst_ and *R*_free_ of 19.1% and 23.0%, respectively. The data collection and refinement statistics are summarized in Table 2. The electron density of residues 136–321 was clearly observed, whereas that of residues 123–135 and 322–324 was not found. The regions that could be clearly visualized in the crystal structure were identical to the previously determined crystal structure of the WT (observed region: 136–321). In the superposition of both structures, the positions of the main and side chains were equivalent (root‐mean‐square deviations: 0.12 Å; Fig. S7).

**Table 2:** Data collection and refinement statistics.

	TTE^Δ100^ (residues 123–324)
Data collection	
X‐ray source	BL45XU
Wavelength (Å)	1.0000
Space group	*I*2_1_2_1_2_1_
Unit cell parameters (Å)	*a* = 63.09, *b* = 106.40, *c* = 117.78
Resolution range (Å)^a^	39.91–2.45 (2.55–2.45)
*R*_merge_ (%)^a^ ^,^ ^b^	17.0 (91.2)
Completeness (%)^a^	99.9 (99.9)
Total reflections^a^	164 936 (18 190)
Unique reflections^a^	14 957 (1651)
Redundancy^a^	11.0 (11.0)
*I*/σ(*I*)^a^	14.3 (3.9)
*CC*_1/2_ ^a^	0.993 (0.840)
Refinement	
Resolution range (Å)^a^	39.94–2.45 (2.514–2.450)
Number of reflections^a^	14 234 (1529)
*R*_cryst_/*R*_free_ (%)^a^ ^,^ ^c^ ^,^ ^d^	19.1 (26.5)/23.0 (29.6)
RMSD bond length (Å)	0.012
RMSD bond angle (°)	1.868
Number of atoms	1529
Protein	1488
Metal	1
Ligand	6
Water	34
Average B‐factor	
Protein	40.5
Ligand	64.1
Water	32.4
Ramachandran plot (%)^e^	
Favored	95.7
Allowed	3.3
PDB code	7Y51

^a^ Values in parentheses are for the highest‐resolution shell.

^b^ *R*_merge_ = Σ_hkl_Σ_i_¦*I*_hkl,j_ − <*I*_hkl_>¦/Σ_hkl_Σ_i_ *I*_hkl,j_, where *I*_hkl,j_ is the intensity of the observation, and *I*_hkl,j_ and <*I*_hkl_> are the averages of the symmetry‐related observations of a unique reflection.

^c^ *R*_cryst_ = Σ¦¦*F*_o_¦ − ¦*F*_c_¦¦/Σ¦*F*_o_¦, where *F*_o_ and *F*_c_ are the observed and calculated structure factor amplitudes, respectively.

^d^ *R*_free_ was calculated using a randomly selected 5% of the dataset, which was omitted from all the stages of refinement.

^e^ Ramachandran plots were prepared for all residues, except glycine and proline.

**Fig. 5:**
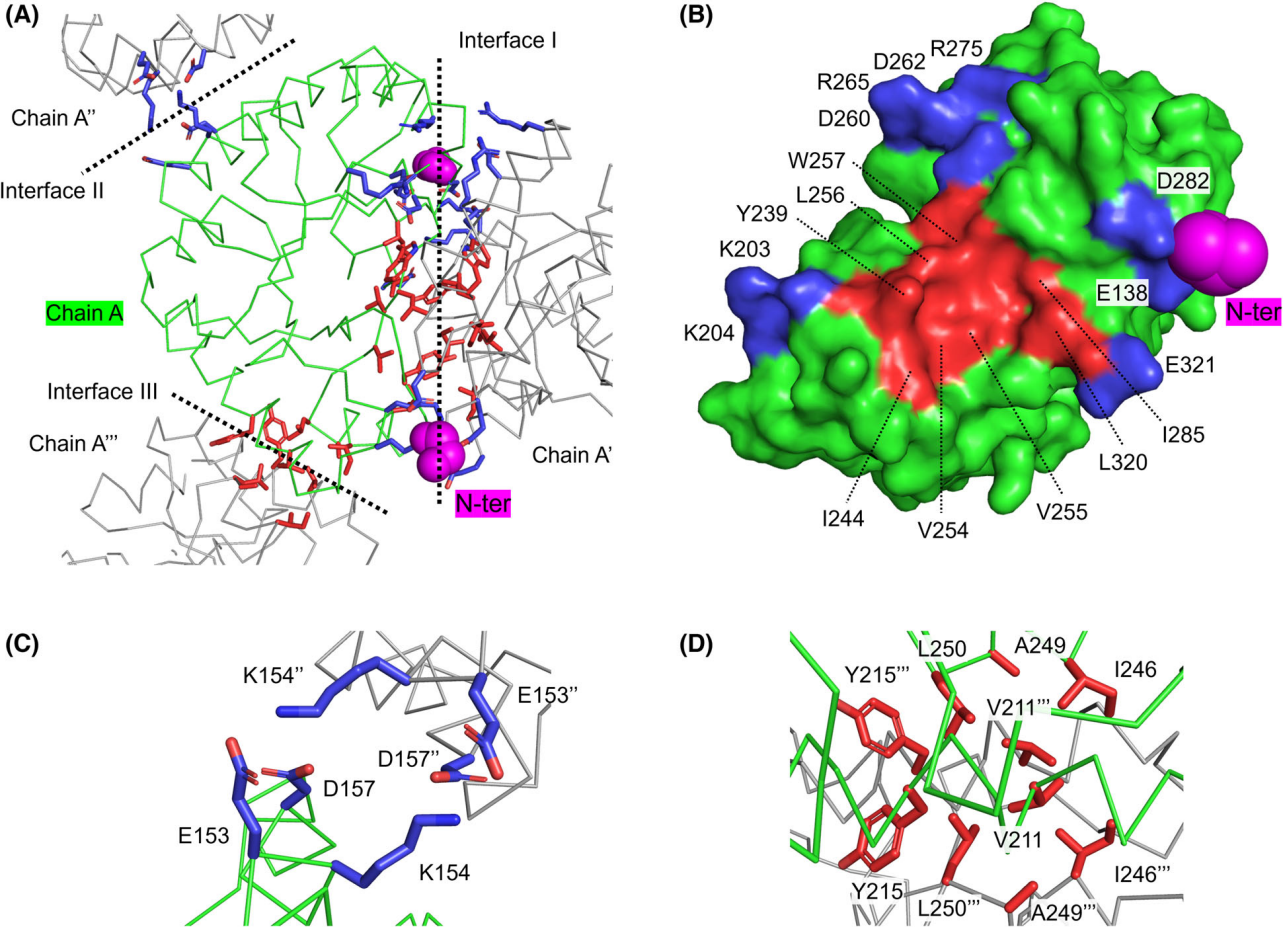
Molecular interaction of TTE^Δ100^. (A) Molecular interfaces of chain A and A', A'', A'''. Molecular interaction of (B) interface I, (C) interface II, and (D) interface III. Chain A is presented as a green ribbon and surface. Chain A', A'', and A''' are presented as a gray ribbon. The N‐terminal amino acid residue (G136) is represented as a magenta sphere. The charged and hydrophobic amino acids are represented as blue and red sticks, respectively.

The molecular interactions of NTR truncation were investigated. Intermolecular contacts in the crystal were visualized using symmetric operation, which displays neighboring symmetric molecules in a crystal lattice. One molecule of the asymmetric unit (chain A) was in contact with the −*x*, −*y* + 1/2 (chain A'), −*y*, −*z* + 1/2 (chain A''), and −*x* + 1/2, −*z* (chain A''') symmetry operation of chain A through interfaces I, II, and III, respectively (Fig. 5A). Interface I was composed of electrostatic and hydrophobic interactions (Fig. 5A,B). Two electrostatic interactions of interface I with residues E138, K203, K204, D282, and E321 were observed around the N‐terminus of both molecules. In addition, electrostatic interactions of interface I were observed among D260, D262, R265, and R275 residues and the same residues of the symmetric molecule for a total of eight residues. The hydrophobic interaction of interface I was observed among Y239, I244, V254, V255, L256, W257, I285, and L320 residues and the same residues of the symmetric molecule for a total of 16 residues. The electrostatic interaction of interface II was observed among the E153, K154, and D157 residues and the same residues of the symmetric molecule for a total of six residues (Fig. 5C). The hydrophobic interaction of interface III was observed among V211, Y215, I246, A249, and L250 residues and the same residues of the symmetric molecule for a total of 10 residues (Fig. 5D).

Among interfaces I, II, and III, interface I had the largest number of residues involved in molecular interactions, with 34 residues. Therefore, interface I was considered to form preferentially and more strongly than the interfaces II and III. SEC results showed that dimerization was observed in TTE^Δ100^ and TTE^Δ110^ with almost truncated NTR, but not in WT to TTE^Δ80^ (Fig. 2C). This suggested that the cleavage of the NTR promoted electrostatic interaction and assembly at interface I to form a hydrophobically interacting dimer (Fig. 5A,B). The symmetric interactions at interfaces II and III promoted the rapid assembly of dimers to form crystals. Although our previous report could not clearly refer to the presence of NTR in the crystals, the present results suggest that NTR was cleaved over a long crystallization time (6 months) and then assembled and crystallized through electrostatic interactions [10]. These results suggest that the NTR functions in way to cover the molecular surface of the catalytic domain and prevent dimerization, causing the rapid formation of protein crystals.

## Conclusion

In this study, we found that NTR was involved in the prevention of crystallization of the catalytic domain in TTE0866. The NTR was identified as an IDR by sequence prediction, secondary structure analysis, and limited proteolysis. The NTR fragment improved the solubility of the NTR‐truncated TTE^Δ100^. As a result of the decreased solubility, TTE^Δ100^ was easily crystallized in a conventional buffer solution without a precipitant, suggesting easy crystallization of the catalytic domain. These findings provide new insights into the role of the NTR region in CE4 proteins.

## Conflict of interest

The authors declare no conflict of interest.

## Author contributions

KS, KU, TN, and YN conceived and designed the project; KS and TH acquired the data; KS, TH, and YN analyzed and interpreted the data; KS, TH, and YN wrote the paper; KS, TH, KM, TO, KU, TN, and YN discussed the results and contributed to the final manuscript.

## Supporting information


**Table S1.** Inverse PCR primers.
**Fig. S1.** NTR amino acid sequence of NTR truncation mutants.
**Fig. S2.** Weak repetitive sequences of NTR.
**Fig. S3.** Temperature dependence of CD signals at 222 nm. Samples: WT (black), TTE^Δ100^ (red), and TTE^Δ110^ (blue).
**Fig. S4.** Protein solubilities of WT and TTE^Δ100^. Protein concentration of WT and TTE^Δ100^ in (A) 20 mm sodium phosphate (pH 6.0) and (B) 20 mm CHES‐NaOH (pH 10.0) containing 0, 50, 100, and 150 mm NaCl. Samples: 100 μm WT (closed circles) and 100 μm TTE^Δ100^ (open circles). Values are presented as the means ± SD (*n* = 3).
**Fig. S5.** SDS/PAGE of TTE^Δ100^ (100 μm) in the presence of NTR fragments (0, 50, 100, 150, and 200 μm) in 20 mm Tris–HCl (pH 8.0). Proteins were visualized using Coomassie brilliant blue.
**Fig. S6.** Crystallization of TTE^Δ100^. Scale bars = 0.1 mm.
**Fig. S7.** Overall structure comparison of WT (PDB ID: 7FBW) and TTE^Δ100^. WT and TTE^Δ100^ are presented as magenta and green cartoons, respectively. The sphere shows Ni^2+^ ion.

## Data Availability

The structural data obtained in this study were deposited in the PDB under the accession code 7Y51.
