# Supplementary material for: Functional analysis of the N‐terminal region of acetylxylan esterase from Caldanaerobacter subterraneus subsp. tengcongensis
Source: FEBS Open Bio. 2022 Sep 20;12(10):1875–85. doi: 10.1002/2211-5463.13476 (PMC9527590; doi:10.1002/2211-5463.13476)
Supplement: Supplementary file 1 — Table S1. Inverse PCR primers. Fig. S1. NTR amino acid sequence of NTR truncation mutants. Fig. S2. Weak repetitive sequences of NTR. Fig. S3. Temperature dependence of CD signals at 222 nm. Samples: WT (black), TTEΔ100 (red), and TTEΔ110 (blue). Fig. S4. Protein solubilities of WT and TTEΔ100. Protein concentration of WT and TTEΔ100 in (A) 20 mm sodium phosphate (pH 6.0) and (B) 20 mm CHES‐NaOH (pH 10.0) containing 0, 50, 100, and 150 mm NaCl. Samples: 100 μm WT (closed circles) and 100 μm TTEΔ100 (open circles). Values are presented as the means ± SD (n = 3). Fig. S5. SDS/PAGE of TTEΔ100 (100 μm) in the presence of NTR fragments (0, 50, 100, 150, and 200 μm) in 20 mm Tris–HCl (pH 8.0). Proteins were visualized using Coomassie brilliant blue. Fig. S6. Crystallization of TTEΔ100. Scale bars = 0.1 mm. Fig. S7. Overall structure comparison of WT (PDB ID: 7FBW) and TTEΔ100. WT and TTEΔ100 are presented as magenta and green cartoons, respectively. The sphere shows Ni2+ ion. [file FEB4-12-1875-s001.docx]

**Supplementary information**

**Table S1.** Inverse PCR primers.

| Name | Sequence (5’ to 3’) |
| --- | --- |
| pET32b rev | CGGACCTTGAAACAGAACTTCCAGGCTGTC |
| TTE^Δ20^ for | GAAAATGTGAATTTAAATACCCTTGACAGTAAATC |
| TTE^Δ40^ for | GAAGAACGTCCTTTAAGCGAAACGGAGCAA |
| TTE^Δ52^ for | GTCTCTTCAACACCAGAACCTTCT |
| TTE^Δ60^ for | ACTCCTGAAAAGGTCTTAGAAAAACATAATAAAGA |
| TTE^Δ80^ for | CAATTTATTCTCAACTTTGTAAACAGGCCTGAAAG |
| TTE^Δ100^ for | GCTTTTAGCAAAAAGGTATTAGGTTCAAATCCTTC |
| TTE^Δ110^ for | CCTTCCTCTGGAAAAGAAGTAGCTTTGACT |


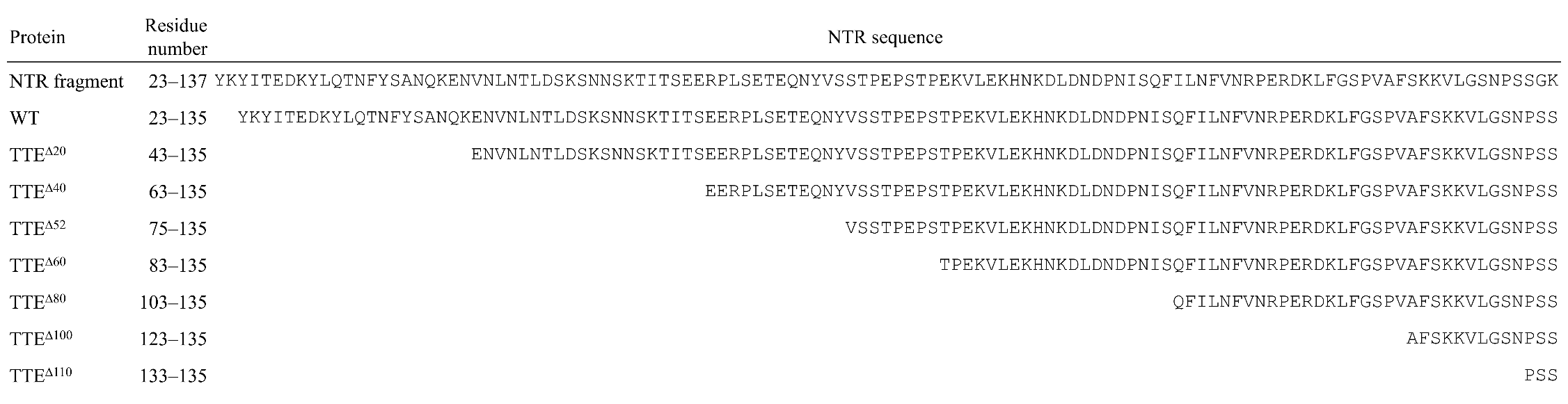
**Fig. S1.** NTR amino acid sequence of NTR truncation mutants.





**Fig. S2.** Weak repetitive sequences of NTR.


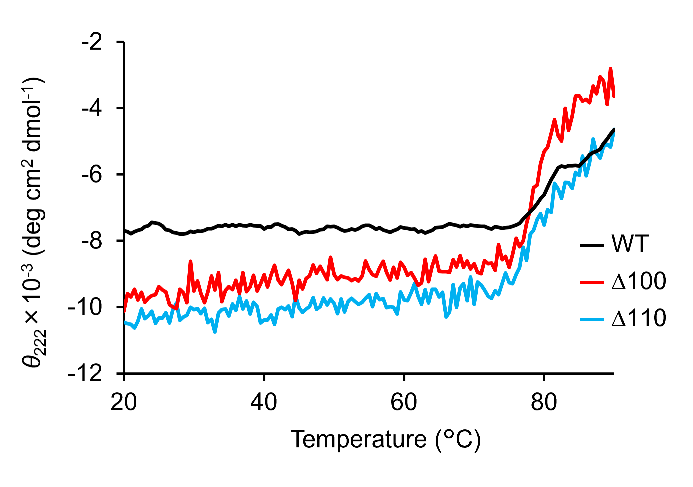


**Fig. S3.** Temperature dependence of CD signals at 222 nm. Samples: WT (black), TTE^Δ100^ (red), and TTE^Δ110^ (blue).


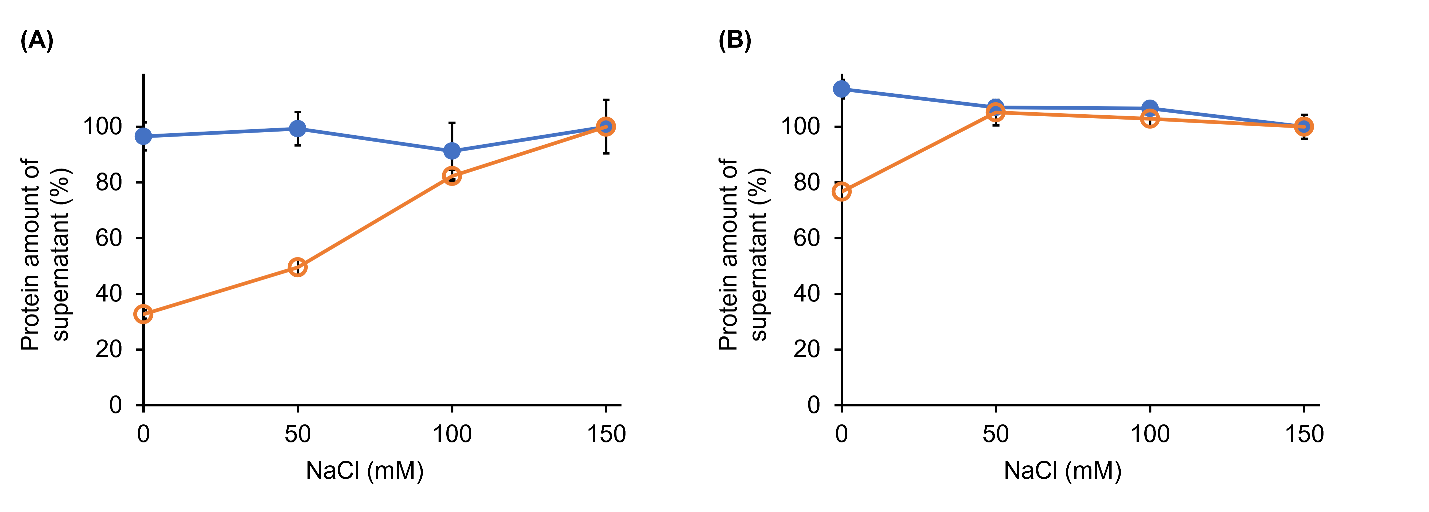
**Fig. S4.** Protein solubilities of WT and TTE^Δ100^. Protein concentration of WT and TTE^Δ100^ in (A) 20 mM sodium phosphate (pH 6.0) and (B) 20 mM CHES-NaOH (pH 10.0) containing 0, 50, 100, and 150 mM NaCl. Samples: 100 µM WT (closed circles) and 100 µM TTE^Δ100^ (open circles). Values are presented as the means ± SD (*n* = 3).


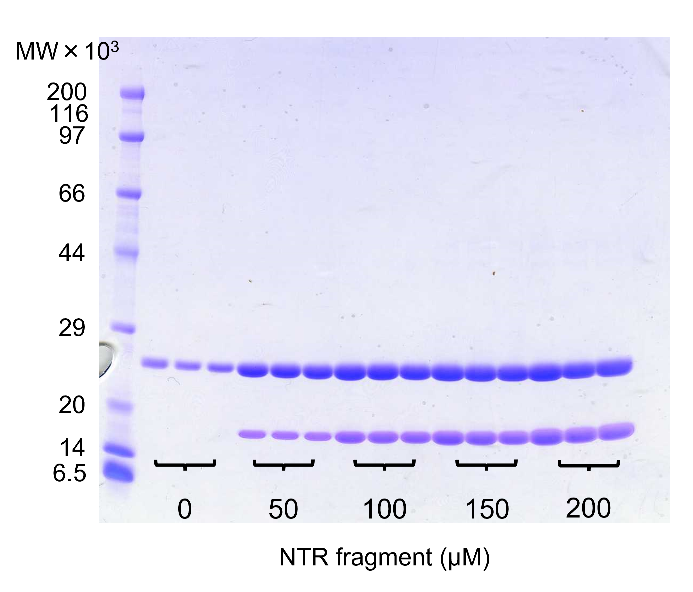


**Fig. S5.** SDS/PAGE of TTE^Δ100^ (100 µM) in the presence of NTR fragments (0, 50, 100, 150, and 200 µM) in 20 mM Tris-HCl (pH 8.0). Proteins were visualized using Coomassie brilliant blue.


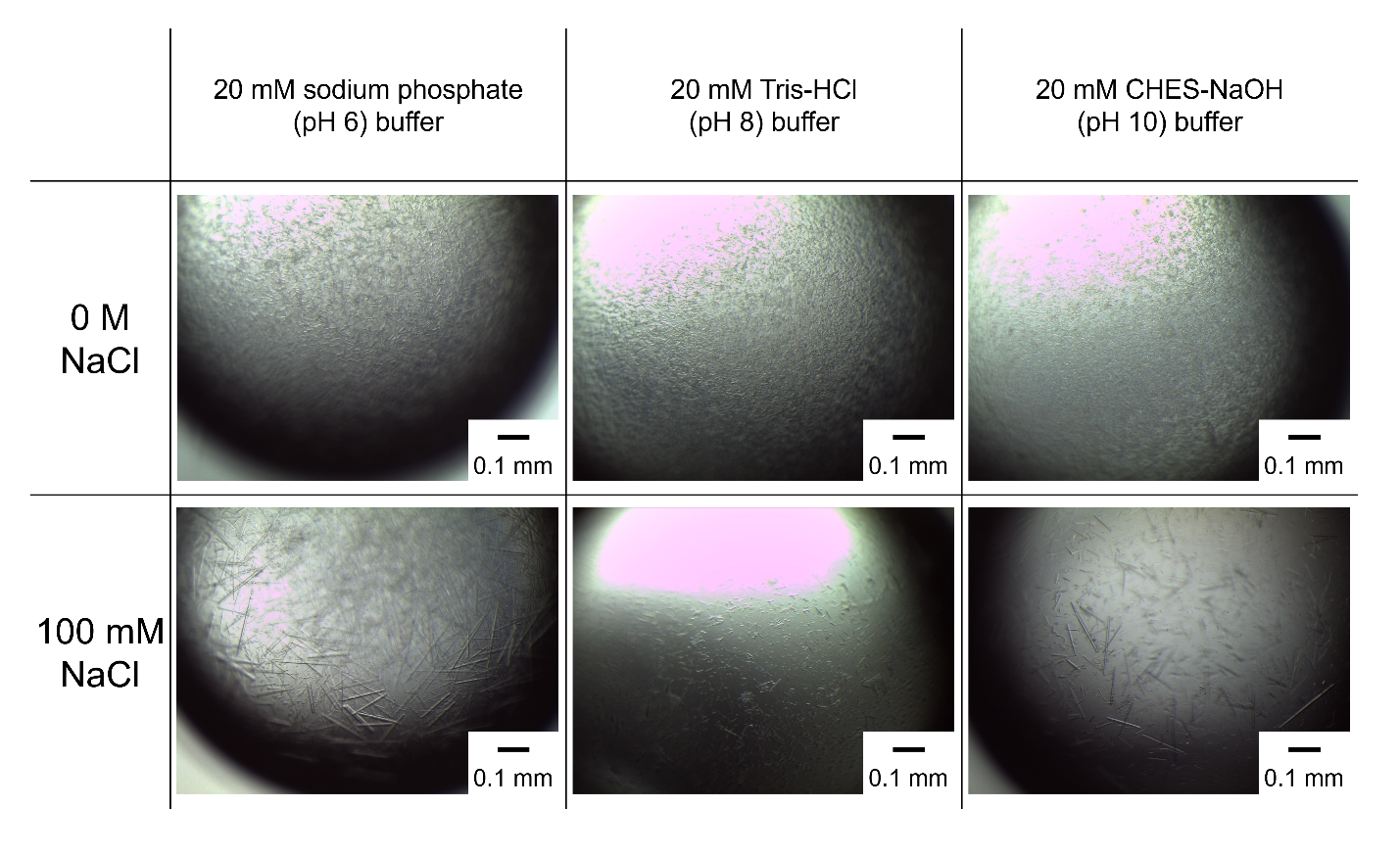
**Fig. S6.** Crystallization of TTE^Δ100^. Scale bars = 0.1 mm.


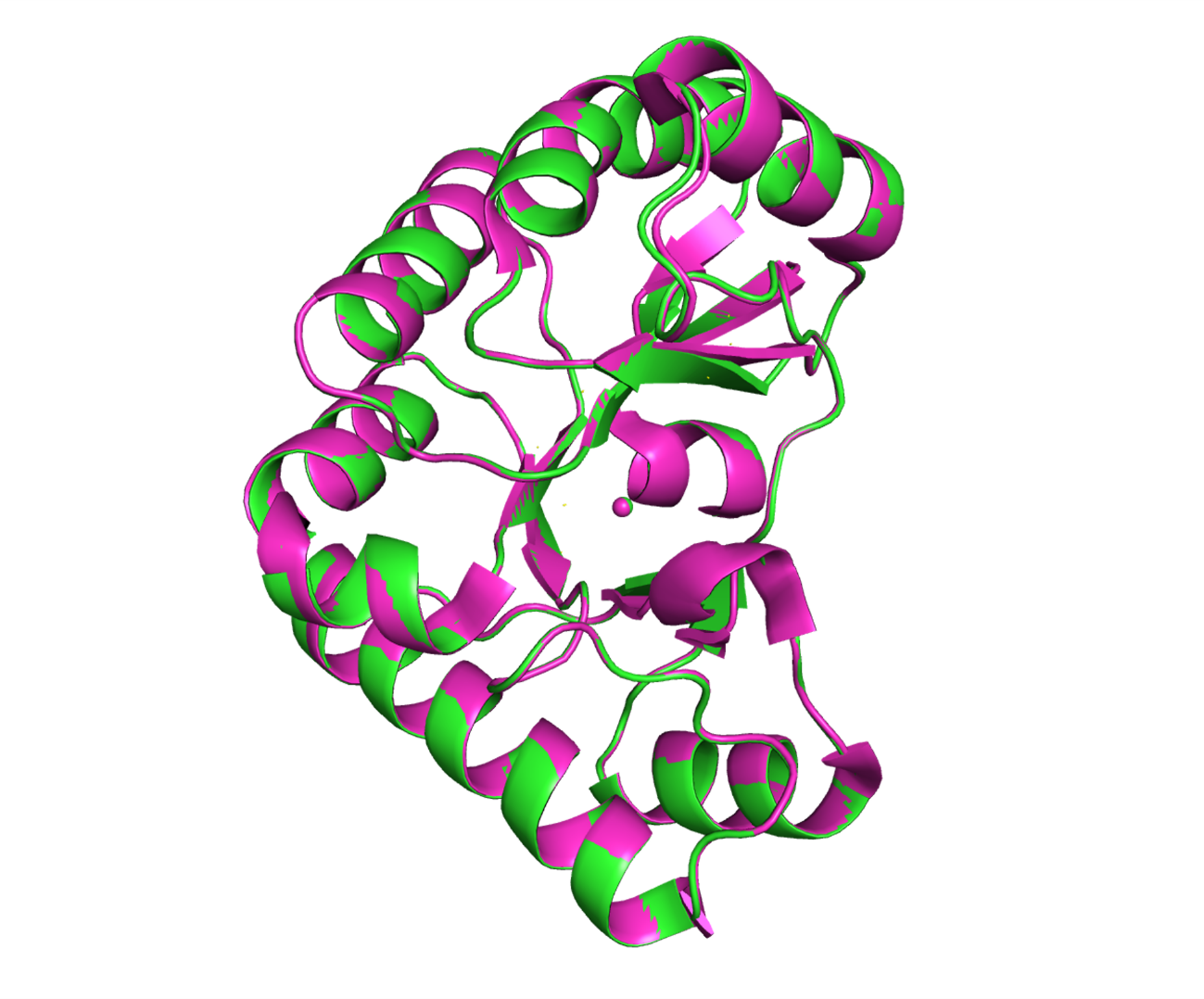


**Fig. S7.** Overall structure comparison of WT (PDB ID: 7FBW) and TTE^Δ100^. WT and TTE^Δ100^ are presented as magenta and green cartoons, respectively. The sphere shows Ni^2+^ ion.
